# Virulence potential of five major pathogenicity islands (SPI-1 to SPI-5) of *Salmonella enterica *serovar Enteritidis for chickens

**DOI:** 10.1186/1471-2180-9-268

**Published:** 2009-12-19

**Authors:** Ivan Rychlik, Daniela Karasova, Alena Sebkova, Jiri Volf, Frantisek Sisak, Hana Havlickova, Vladimir Kummer, Ariel Imre, Annamaria Szmolka, Bela Nagy

**Affiliations:** 1 Veterinary Research Institute, Hudcova 70, 621 00 Brno, Czech Republic; 2 Veterinary Medical Research Institute of the Hungarian Academy of Sciences, Hungaria krt. 21, 1143 Budapest, Hungary

## Abstract

**Background:**

*Salmonella *is a highly successful parasite of reptiles, birds and mammals. Its ability to infect and colonise such a broad range of hosts coincided with the introduction of new genetic determinants, among them 5 major pathogenicity islands (SPI1-5), into the *Salmonella *genome. However, only limited information is available on how each of these pathogenicity islands influences the ability of *Salmonella *to infect chickens. In this study, we therefore constructed *Salmonella *Enteritidis mutants with each SPI deleted separately, with single individual SPIs (i.e. with the remaining four deleted) and a mutant with all 5 SPIs deleted, and assessed their virulence in one-day-old chickens, together with the innate immune response of this host.

**Results:**

The mutant lacking all 5 major SPIs was still capable of colonising the caecum while colonisation of the liver and spleen was dependent on the presence of both SPI-1 and SPI-2. In contrast, the absence of SPI-3, SPI-4 or SPI-5 individually did not influence virulence of *S*. Enteritidis for chickens, but collectively they contributed to the colonisation of the spleen. Proinflammatory signalling and heterophil infiltration was dependent on intact SPI-1 only and not on other SPIs.

**Conclusions:**

SPI-1 and SPI-2 are the two most important pathogenicity islands of *Salmonella *Enteritidis required for the colonisation of systemic sites in chickens.

## Background

*Salmonella *diversified from a common ancestor with *E. coli *approx. 100 million years ago [1]. This diversification was associated with the acquisition of genes which increased the virulence of *Salmonella *and enabled it to interact with its hosts and colonise the intestinal tract of animals in a different way than *E. coli *did. The genomic sequences of *E. coli *and *S*. *enterica *serovars Typhi and Typhimurium have been known since 1997 and 2001, respectively [2-4] and genes which are absent in *E. coli *and are necessary for the full virulence expression of *Salmonella *are therefore relatively well described. Most of them are clustered at specific parts of the *Salmonella *chromosome called pathogenicity islands. There are 5 major pathogenicity islands in the *Salmonella enterica *chromosome but only 4 of them, with SPI-2 absent, in the chromosome of *Salmonella bongori*, a second species belonging to the genus *Salmonella *[5].

The major pathogenicity islands include SPI-1, SPI-2, SPI-3, SPI-4 and SPI-5. The SPI-1 and SPI-2 genes code for proteins forming the type III secretion system (T3SS) which enable the transport of *S. enterica *proteins from the bacterial cell directly into the cytosol of eukaryotic cells. The SPI-1 encoded T3SS is required for the transport of *S. enterica *proteins across the cytoplasmic membrane of a host cell into its cytosol where they induce cytoskeletal rearrangements resulting in the uptake of *S. enterica *even by non-phagocytic cells [6]. In addition, it has been reported that SPI-1 genes, independent of cell invasion, induce macrophage cytotoxicity [7]. Interestingly, neither of these functions is required for the *S*. Typhimurium virulence for Balb/C mice since a mutant without the whole SPI-1 was as virulent as the control wild type strain [8]. SPI-2 encoded T3SS is required for the transport of *S. enterica *proteins across the phagosomal membrane and increases *S. enterica *survival inside phagocytic cells [9,10]. The function of genes localised on the remaining SPIs is less well characterised; SPI-3 genes are involved both in gut colonisation due to MisL-dependent fibronectin binding and intracellular survival due to high-affinity magnesium transport encoded by *mgtABC *[11,12]. SPI-4 genes are required for the intestinal phase of disease by coding for non-fimbrial adhesin [13], and the genes localised in SPI-5 are co-regulated with either SPI-1 or SPI-2 genes and therefore code for effector proteins transported by either of these T3SS [14]. However, the vast majority of this information has been obtained in a mouse model and *S*. Typhimurium and much less data are available for *S*. Enteritidis and pigs, cattle or poultry although these animal species, and poultry in particular, represent major reservoirs of *Salmonella *for the human population in Europe.

The roles of different SPI genes in the virulence *S. enterica *for chickens are less well understood. Similarly to mice, the importance of SPI-2 for *Salmonella *persistence in the internal organs has been described in chickens [15-17]. However, unlike the situation in mice, it seems that in chickens, SPI-1 genes are required for both the colonisation of the intestinal tract and the ability to reach and persist in internal organs such as the liver and spleen [17-19]. The importance of the other SPIs for *Salmonella *virulence in chickens is even less clear. To our knowledge, SPI-3 mutants have not been tested in chickens at all, SPI-4 mutants have been tested and shown to have no effect on chicken gut colonisation [13] and SPI-5 genes, although involved in the induction of the proinflammatory immune response in cattle, have been described as having no significant function in chickens [13,20].

In this study we therefore compared virulence of isogenic mutants of *S. enterica *subsp. *enterica *serovar Enteritidis (*S*. Enteritidis) defective in 5 major pathogenicity islands for day-old chickens. To do this we deleted SPI-1 to SPI-5 from the *S*. Enteritidis chromosome and orally infected chickens with these mutants. Our data indicate that the colonisation of the liver and spleen by *S*. Enteritidis in chickens is dependent on SPI-1 and SPI-2 and that the remaining SPIs individually have no effect on *S*. Enteritidis virulence although collectively they had a low effect on spleen colonisation.

## Results

### Infection of chickens - colonisation of the caecum, liver and spleen

**Figure 1:**
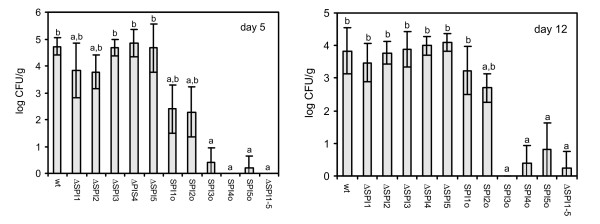
**Distribution of *S*. Enteritidis 147 wild-type strain and SPI mutants in the spleen of orally infected chickens**. *S*. Enteritidis counts in the liver correlated with counts in the spleen except for the fact that ΔSPI2 mutant colonised liver significantly less efficiently than the wild type *S*. Enteritidis also on day 12 (not shown). Y axis, average log CFU/g of spleen ± SD. a, b - ANOVA different at p < 0.05 in comparison to the group infected with the wild-type *S*. Enteritidis (a) or ΔSPI1-5 mutant (b). Abbreviations: wt - wild-type *S*. Enteritidis 147; ΔSPI1-5: mutant from which all major 5 SPI have been removed, ΔSPI1, ΔSPI2, ΔSPI3, ΔSPI4, ΔSPI5: mutants from which the respective SPI has been removed, SPI1o, SPI2o, SPI3o, SPI4o, SPI5o: "SPIonly" mutants, mutants with only the respective SPI retained.

Both on day 5 and day 12, no significant differences in caecal colonisation were observed amongst all the mutants (data not shown). When the ability to persist in internal organs was analysed, the mutants could be clustered into 3 different groups as summarised in Table 1. The first group consisted of the wild-type strain and the ΔSPI3, ΔSPI4 and ΔSPI5 mutants. These strains colonised the liver and spleen with equal efficiency. The second group was formed by ΔSPI1-5, and the SPI3o, SPI4o and SPI5o mutants characterised by their inability to reach and persist in the liver and spleen of chickens. The last group was formed by ΔSPI1, ΔSPI2, and the SPI1o and SPI2o mutants which exhibited an intermediate ability to persist in liver and spleen of infected chickens (Fig. 1).

**Table 1:** *S*. Enteritidis 147 and its SPI mutants grouped according to their ability to colonise the liver and spleen of one-day-old chickens

Group 1	Group 2	Group 3
**virulent**	**avirulent**	**medium virulent**

wt	ΔSPI1-5	ΔSPI1
ΔSPI3	SPI3o	ΔSPI2
ΔSPI4	SPI4o	SPI1o
ΔSPI5	SPI5o	SPI2o

wt - wild-type *S*. Enteritidis 147; ΔSPI1-5: mutant from which all major 5 SPI have been removed; ΔSPI1, ΔSPI2, ΔSPI3, ΔSPI4, ΔSPI5: mutants from which the respective SPI has been removed; SPI1o, SPI2o, SPI3o, SPI4o, SPI5o: mutants with only the respective SPI retained

**Figure 2:**
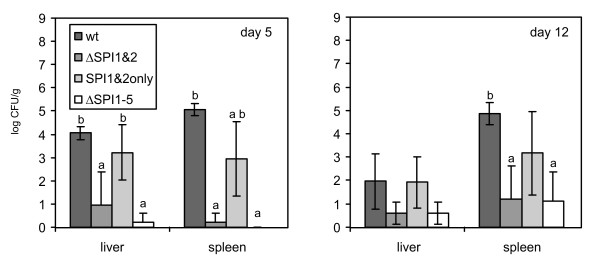
**Distribution of *S*. Enteritidis 147 wild-type strain and ΔSPI1&2 and SPI1&2o, ΔSPI1-5 mutants in the liver and spleen of orally infected chickens**. Y axis, average log CFU/g of organ ± SD. a, b - *t*-test different at p < 0.05 in comparison to the group infected with the wild-type *S*. Enteritidis (a) or the ΔSPI1-5 mutant (b). Abbreviations: wt - wild-type *S*. Enteritidis 147; ΔSPI1-5: mutant from which all major 5 SPIs have been removed; ΔSPI1&2: mutant from which SPI1 and SPI2 have been removed; SPI1&2 only: mutant with only SPI1 and SPI2 retained.

The above-mentioned data indicated that SPI-1 and SPI-2 were the two major pathogenicity islands required for chicken colonisation. To verify this, in the next step we constructed two additional mutants - the first one without both the SPI-1 and SPI-2 (ΔSPI1&2 mutant) and the second one with only the SPI-1 and SPI-2 retained (SPI1&2o mutant), and we repeated the infections including the wild-type *S*. Enteritidis strain and *S*. Enteritidis ΔSPI1-5 mutant as controls. The presence of only these two SPIs allowed the SPI1&2o mutant to colonise the liver almost as efficiently as did the wild-type strain although this mutant exhibited a minor defect in spleen colonisation indicating the cumulative influence of SPI-3, SPI-4 and SPI-5 on the spleen-colonising ability of *S*. Enteritidis. The defect could be observed both on day 5 and day 12 although a statistically significant difference from the both the wild type strain and the ΔSPI1-5 mutant infected chickens could be detected only on day 5. On the other hand, the mutant without these 2 SPIs behaved exactly as the ΔSPI1-5 mutant and was only rarely recovered from the liver and spleen (Fig. 2).

### Histology in chickens

**Figure 3:**
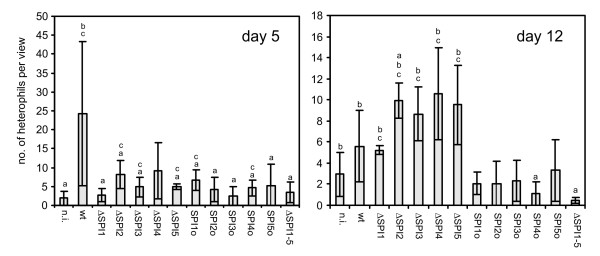
**Heterophil infiltration in caeca of chickens infected with different SPI mutants of *S*. Enteritidis**. Y axis, average number of heterophils per microscopic view ± SD. a, b, c - ANOVA test different at p < 0.05 in comparison to the group infected with the wild-type *S*. Enteritidis (a), the ΔSPI1-5 mutant (b), or the non-infected controls (c). Abbreviations: as in Fig. 1.

Histological examination revealed no differences in the livers of chickens infected with any of the mutants or with the wild-type strain. On the other hand, different degrees of inflammation and heterophil infiltration were found in the caeca on day 5, and this infiltration was dependent on the presence of SPI-1. The ΔSPI1 mutant was the only single SPI deletion mutant which induced significantly less heterophil infiltration than the wild-type *S*. Enteritidis, and chickens infected with this mutant did not differ from those infected with the ΔSPI1-5 or the non-infected chickens (Fig. 3). By day 12, none of the SPI 'only' mutants stimulated heterophil infiltration any longer, and also the heterophil infiltration in chickens infected with the ΔSPI1 mutant remained at quite a low level. In chickens infected with the wild-type strain, heterophil infiltration dropped between day 5 and day 12 and heterophil infiltration induced by the wild type strain on day 12 was similar to that induced by the ΔSPI1 mutant (Fig. 3).

### Proinflammatory cytokine response

Previous experiments had shown that the early heterophil infiltration decreased with the loss of SPI-1. We therefore tested cytokine signalling in the caeca of chickens infected with the ΔSPI1, ΔSPI2 and ΔSPI1&2 mutants. For all the cytokines measured, an identical trend was observed - the highest induction was observed in chickens after infection with the wild type strain, followed by those infected with ΔSPI2, ΔSPI1 and ΔSPI1&2 mutants, respectively (data not shown). Except for IL-12β, the expression of the remaining cytokines after infection with the wild-type strain and the ΔSPI2 mutant significantly differed from the expression observed in non-infected control chickens while the differences between the non-infected chickens and those infected with the ΔSPI1 and ΔSPI1&2 mutant were always insignificant.

## Discussion

In this study we were interested in the role of five major pathogenicity islands in the virulence of *S*. Enteritidis for chickens. Rather unexpectedly, none of the pathogenicity islands was essential for colonisation of the intestinal tract despite the fact that other studies demonstrated that single gene SPI-1 mutants in chickens or SPI-4 mutants in cattle showed impaired intestinal colonisation and/or mucosa invasion [13,18]. We cannot exclude the possibility that, if the infectious dose was changed or the duration of animal infection was extended for a longer period of time, we would observe a correlation between the persistence in the gut and the presence of a particular SPI. It is also possible that the differences between a single gene mutant and the whole SPI-1 mutant are biologically relevant because in mice a difference in the behaviour of the whole SPI-1 mutant and a *hilA *mutant was observed. This difference has been explained by the presence of the SPI-1 localised genes stimulating the host's immune response, the effect of which is suppressed in the presence of intact *hilA *[8]. Finally, it is also possible that a low-level expression of SPI-1 encoded T3SS, which is likely to occur in the *hilA *mutant, might be still high enough to induce the interference with some of the eukaryotic host cell function(s) resulting in a ΔSPI1 mutant phenotype different from that of single gene mutants used in the above-mentioned other studies.

Although no influence of SPIs on gut colonisation was observed, SPI-1 and SPI-2 pathogenicity islands were both required for *S*. Enteritidis colonisation of the liver and spleen, similar to previous studies [9,13,18,21]. Interestingly, the decrease in counts of the ΔSPI1 and ΔSPI2 mutants in the liver and spleen was numerically not as high as that observed for single gene SPI-2 mutants in mice [22]. The importance of these two SPIs for *S*. Enteritidis colonisation of the liver and spleen of chickens was further supported by the behaviour of SPI1o and SPI2o mutants which, when compared with the ΔSPI1-5 mutant, had a significantly higher ability to colonise the spleen of infected chicken, and also by the ΔSPI1&2 mutant which did not differ in colonisation of liver and spleen from the ΔSPI1-5 mutant. Interestingly, the deletion of SPI-1 resulted in a significant difference from the wild type strain liver colonisation on day 5 but not on day 12 in agreement with the results of Desin et al. [19] suggesting that decreased liver colonisation by the ΔSPI1 mutant might be caused by its slower translocation through the gut epithelium. On the other hand, the ΔSPI2 mutant showed decreased liver colonisation both on day 5 and day 12 when compared with the wild-type strain, which is consistent with the role of SPI-2 encoded proteins in intra-macrophage survival [10]. The importance of SPI-1 and SPI-2 was further confirmed by the virulence of SPI1o and SPI2o mutants because the presence of each of these pathogenicity islands individually increased the virulence of *S*. Enteritidis for chickens. Our observations on SPI-1 and SPI-2 as the most important SPIs are similar to those of Dieye et al. except for the fact that we could not confirm that SPI-1 would be more important than SPI-2 for *Salmonella *infection of chickens [17] although we did observe that SPI-1 was the most important for the induction of inflammation as supported by the cytokine inductions and the influx of heterophils. Interestingly, unlike the bovine and murine models [23,24], we did not observe any correlation between the absence of SPI-2 and the induction of proinflammatory or any other cytokines in the avian caeca. Furthermore, we did not observe any effect of SPI-3, SPI-4 and SPI-5 deletions on the virulence of *S*. Enteritidis for chickens. This agrees with the observations of Morgan et al. who showed that SPI-4 genes were superfluous and SPI-3 genes and the *pipB *gene of SPI-5 played only a minor role in the colonisation of the chicken gut by *S*. Typhimurium [13]. However since the SPI1&2o mutant showed reduced ability to colonise the spleen 4 days post infection when compared with the wild-type *S*. Enteritidis infection, this shows that SPI-3, SPI-4 and SPI-5 collectively influenced the virulence of *S*. Enteritidis for chickens although these 3 SPIs individually did not contribute to the ability of *S*. Enteritidis to colonise the spleen of infected chickens.

## Conclusions

In this study we have shown that SPI-1 and SPI-2 pathogenicity islands are central to the virulence of *S*. Enteritidis for chickens. The presence of either of these two pathogenicity islands resulted in a significant increase in the liver and spleen colonisation by *S*. Enteritidis. The remaining three major pathogenicity islands (SPI-3, SPI-4 and SPI-5) influenced *S*. Enteritidis virulence for day-old chickens collectively but not individually.

## Methods

### Bacterial strains and culture conditions

*S*. Enteritidis strain 147 was used throughout the study [25]. A clone spontaneously resistant to nalidixic acid was propagated in LB broth supplemented with ampicillin, chloramphenicol or kanamycin if necessary.

### Construction and characterisation of SPI deletion mutants

SPI-5 was removed from the *S*. Enteritidis genome using the λ Red recombination as described [26]. For the construction of the remaining SPI mutants, a modified procedure of λ Red recombination was used. The modification was used because we had failed to remove a sequence greater than 10 kb by a single-step procedure in *S*. Enteritidis 147. We therefore first introduced the chloramphenicol gene cassette at the left end of the sequence to be removed by the standard protocol and in the next step, a kanamycin gene cassette was inserted at the right end of the sequence to be removed. In the case of SPI-1 removal, the chloramphenicol gene cassette was used for the replacement of the *avrA *gene and then the kanamycin gene cassette was used for the replacement of the *invH *gene. The intermediate *avrA*::Cm *invH*::Kan mutant was transformed with pCP20 and any sequence in between the *frt *sequences was removed by pCP20-encoded flipase. Originally we expected to obtain two constructs, ΔSPI1 and SPI1::Cm (or SPI1::Kan), the latter being suitable for transduction. However, since all the mutants recovered were ΔSPI-1, free of any antibiotic resistance marker, to obtain SPI1::Cm (or SPI1::Kan) mutation suitable for transduction, we inserted chloramphenicol or kanamycin resistance gene cassettes into the ΔSPI1 mutant once more using a PCR product resulting from the amplification of pKD3 or pKD4 plasmid template with avrA44For and invH44Rev primers. Using this protocol, we constructed strains in which SPI-1, SPI-2, SPI-3, SPI-4 or SPI-5 were replaced with either chloramphenicol or kanamycin resistance gene cassettes. All the primers used for SPI removal are listed in Table 2.

**Table 2:** List of primers used for the generation and verifications of SPI mutants in *S*. Enteritidis.

SPI deletion	Primer *	Sequence 5' - 3'
SPI1	avrA_44F	TTATCGTTTAGCATAACGGCATTGTTATCGAATCGCTCATAAAGGTGTAGGCTGGAGCTGCTTC
SPI1	avrA_44R	ATGATATTTTCGGTGCAGGAGCTATCATGTGGAGGGAAAAGTATCATATGAATATCCTCCTTAG
SPI1	avrA_FCTR	AGACTTATATTCAGCTATCC
SPI1	avrA_RCTR	ACATAACCCTGCTGTACCTG
SPI1	invH_44Fw	AATATGAAAAAATTTTATAGCTGTCTTCCTGT CTTTTTACTGATCTTCGGAATAGGAACTTCAT
SPI1	invH_R44	ATGAGTTGCTCTTCATCTTCTTTCGAACGCAT GTATTGTGGATGCATATGAATATCCTCCTTAG
SPI1	invH_FCTR	CAGGAGTTTTTTTTGCTAGC
SPI1	invH_RCTR	CATGGGCAGCAAGTAACGTC
SPI2	STM1379_44F	TCAATCGAGCAACTTTTTGCCTTCCAGGTCGATGGCGATGTTTTGTGTAGGCTGGAGCTGCTTC
SPI2	STM1379_44R	ATCATGAAAAAAGTCAAAAAATTGTCTCTTACCGATTTAGTGCTCATATGAATATCCTCCTTAG
SPI2	STM1379_FCTR	ACCATTCAAGAGACAATTGG
SPI2	STM1379_RCTR	GTCCTGTTCTGGTACTACGC
SPI2	ssaU_44Fw	ATGAGCGAGAAAACAGAACAGCCTACAGAAAAGAAATTACGTGAACTTCGGAATAGGAACTTCA
SPI2	ssaU_44R	TTATGGTGTTTCGGTAGAATGCGCATAATCTA TCTTCATCACCACATATGAATATCCTCCTTAG
SPI2	ssaU_FCTF	TATGGTATTAGCCGATCTGG
SPI2	ssaU_RCTR	ACCTTTATCGTCAAGCACTG
SPI3	STM3752_44F	TCAACGTATAGAGCCATCCGGATAAAGATACATGCCTCCCTCCAGTGTAGGCTGGAGCTGCTTC
SPI3	sugR_44R	TCTTCATGCGGCGGCTGTTCTCCTCGCTTGTC GAGCATCCAGGTCATATGAATATCCTCCTTAG
SPI3	STM3752_FCTR	GGATATCGTCTGCAAAGAAG
SPI3	sugR_RCTR	CTAGATGTTCACGGTAGCTA
SPI3	mgtC_44Fw	TTATTGACTATCAATGCTCCAGTGAATTGCGGTGATATTATCGTACTTCGGAATAGGAACTTCA
SPI3	mgtC_44R	CCAGAAAAAATGGAGGAACGTATGTTAATGTTTCCTTATATTTACATATGAATATCCTCCTTAG
SPI3	mgtC_FCTR	ATGAATCCCCAAAATTAAGG
SPI3	mgtC_RCTR	AATCATCTGGCAAGTTAACG
SPI4	STM4257_44F	ATGGAAGACGAAAGTAATCCGTGGCCTAGTTTTGTTGATACATTGTGTAGGCTGGAGCTGCTTC
SPI4	STM4257_44R	TCACTCTGACACCTTTTTATTAATAGTCGTGATAATAGCTTTAC CATATGAATATCCTCCTTAG
SPI4	STM4257_FCTR	TAAAGCGTATTGGTAGCAGG
SPI4	STM4257_RCTR	TAATGCACACAAAGAACCTG
SPI4	STM4262_44Fw	CGTATAGCCGATATTCCAATATTTATTATATT TCTCATTGTTATACTTCGGAATAGGAACTTCA
SPI4	STM4262_44R	TAAACTCATCTAAGTTATCAAAAATTTGCTTCTCGGTATTCTCACATATGAATATCCTCCTTAG
SPI4	STM4262_FCTR	CAGTCTATCACAGCAAGGCA
SPI4	STM4262_RCTR	TTATCCGGAGAACAATCACG
SPI5	SPI5_44F	ATATCGGGGAAAACAGGTGTATCTGCGGTATT TAATCTATATGTGTGTAGGCTGGAGCTGCTTC
SPI5	SPI5_44R	GAAGATAAAACGATGCAAAATGCGCAGACGCT CGCCCGTCGCCTCATATGAATATCCTCCTTAG
SPI5	SPI5_FCTR	ATATGCGTAACTCATCAGTC
SPI5	SPI5_RCTR	GCTGCAAACGCTGGTTATGC

* Primers "...44..." were used for amplification with pKD3 or pKD4 plasmid templates. Sequence used for annealing with the plasmids is underlined. Primers "...CTR..." were used in control PCRs, forward always with c1 or k1, and reverse always with c2 or k2.

Since the procedure described above required multiple recombination events, finally we transferred the mutations into a fresh host by P22 phage mediated transduction. In the case of multiple SPI mutants we first transduced two mutations enabling antibiotic resistance-based selection into the wild-type strain (e.g. SPI1::Cm and SPI2::Kan). In the next step the antibiotic resistances were removed by transient transformation with pCP20 and two additional mutations (e.g. SPI3::Cm and SPI4::Kan) were introduced by transduction and the antibiotic resistance gene cassettes were removed again by pCP20 encoded flipase, and into such a mutant, the SPI5::Cm cassette was finally transduced and the chloramphenicol resistance gene cassette was removed. The final mutants used in all the experiments were therefore transductants free of any antibiotic resistance except for the original resistance to nalidixic acid of the wild-type strain (see also Table 3 for the list of strains). After the construction, all the mutants were tested by PCR for negative amplification of internal SPI genes and for positive amplification using primers flanking individual SPIs. After the construction, all the mutants remained sensitive to P22 and did not show any obvious defects when grown in nutrient rich LB medium or glucose minimal medium. The mutants were also as resistant as the wild-type strain to the action of blood serum, egg white, bile salts, polymyxin (as a representative of antimicrobial peptides), hydrogen peroxide or pH 4 (not shown).

**Table 3:** List of strains used in this study.

Strain	SPI present	SPI absent	Reference
*S*. Enteritidis 147 Nal wild type	1, 2, 3, 4, 5	none	[25]
*S*. Enteritidis 147 Nal ΔSPI1	2,3,4,5	1	this study
*S*. Enteritidis 147 Nal ΔSPI2	1,3,4,5	2	this study
*S*. Enteritidis 147 Nal ΔSPI3	1,2,4,5	3	this study
*S*. Enteritidis 147 Nal ΔSPI4	1,2,3,5	4	this study
*S*. Enteritidis 147 Nal ΔSPI5	1,2,3,4	5	this study
*S*. Enteritidis 147 Nal ΔSPI1-5	none	1,2,3,4,5	this study
*S*. Enteritidis 147 Nal SPI1o	1	2,3,4,5	this study
*S*. Enteritidis 147 Nal SPI2o	2	1,3,4,5	this study
*S*. Enteritidis 147 Nal SPI3o	3	1,2,4,5	this study
*S*. Enteritidis 147 Nal SPI4o	4	1,2,3,5	this study
*S*. Enteritidis 147 Nal SPI5o	5	1,2,3,4	this study
*S*. Enteritidis 147 Nal ΔSPI1&2	3,4,5	1,2	this study
*S*. Enteritidis 147 Nal SPI1&2o	1,2	3,4,5	this study

### Infection of chickens

In the first experimental infection, day-old chickens (Ross breed, 10 birds/group) were infected orally with 5 × 10^7 ^CFU of either the wild-type strain or the SPI mutants. In the second infection, four groups, each of 10 chickens, were infected with the wild type strain, or ΔSPI1&2, SPI1&2o and SPI1-5 mutants. Counts of the strains in caeca, liver and spleen were determined in 5 birds on day 5 and in remaining 5 birds on day 12 of life i.e. 4 and 11 days post infection, respectively. The last experimental infection was focused on cytokine signaling and in this case, besides 3 non-infected control chickens, three additional chickens per group were infected with wild type strain, ΔSPI1, ΔSPI2, and ΔSPI1&2 mutants. In all euthanised birds, *S*. Enteritidis counts in the caeca, liver and spleen were determined after tissue homogenisation in peptone water and plating tenfold serial dilutions on XLD, BGA or Bromothymol-blue agars (Merck) supplemented with nalidixic acid. Samples negative after the direct plating were subjected to pre-enrichment in RV broth supplemented with nalidixic acid for qualitative *S*. Enteritidis determination. Counts of *S*. Enteritidis positive after the direct plating were logarithmically transformed. In the case of samples positive only after the pre-enrichment, these were assigned a value of 1 and the negative samples were assigned a value of 0. Samples from the caeca and liver were also fixed in 10% formaldehyde and subjected to haematoxylin and eosin staining. Each sample was blindly evaluated for general pathology with particular attention given to the infiltration of the caecal wall with heterophils by determining the average number of these cells per 20 independent microscopic fields. All the animal infections were performed according to the relevant national legislation and were approved and supervised by the Institutional Ethics Committee on Animal Experiments of Veterinary Medical Research Institute of Hungarian Academy of Sciences followed by the approval of the Veterinary and Food Control Station, Budapest, Hungary, and the Institutional Ethics Committee on Animal Experiments of Veterinary Research Institute Brno followed by the approval of the Animal Welfare Committee at the Ministry of Agriculture of the Czech Republic.

### Real-time PCR cytokine quantification

RNA was extracted from the ceacal wall samples stored in RNA Later at -20°C using the RNeasy Lipid Tissue Kit (Qiagen). The purified RNA was eluted in 50 μl RNase-free water and used immediately as a template for reverse transcription using M-MLV reverse transcriptase (Invitrogen) and oligo-T primers. The resulting cDNA was purified by the QIAPrep PCR Purification kit (Qiagen) and used as a template for quantitative PCR. mRNA expression rates of chicken cytokines and immune-relevant proteins IL-8, TNFα, IL-12β, IL-18, iNOS and IFNγ were determined using the QuantiTect™ SYBR^® ^Green RT-PCR Kit (Qiagen) using GAPDH mRNA as a reference. Primer sequences are given in Table 4.

**Table 4:** List of primers used for the quantification of chicken cytokines after the infection with *S*. Enteritidis.

Primer	Sequence 5' - 3'	Product size (bp)	Reference
IL-8For	ATGAACGGCAAGCTTGGAGCT	94	this study
IL-8Rev	GCAGCTCATTCCCCATCTT		
TNFαFor	AATTTGCAGGCTGTTTCTGC	112	this study
TNFαRev	TATGAAGGTGGTGCAGATGG		
IL-12βFor	TGGTCCACGCTTTGCAGAT	140	[25]
IL-12βRev	AAGGTTAAGGCGTGGCTTCTTA		
IL-18For	ACGTGGCAGCTTTTGAAGAT	88	this study
IL-18Rev	GCGGTGGTTTTGTAACAGTG		
iNOSFor	GAACAGCCAGCTCATCCGATA	103	[25]
iNOSRev	CCCAAGCTCAATGCACAACTT		
IFNγFor	GCCGCACATCAAACACATATCT	207	[25]
IFNγRev	TGAGACTGGCTCCTTTTCCTT		
GAPDHFor	GTCAGCAATGCATCGTGCA	102	[25]
GAPDHRev	GGCATGGACAGTGGTCATAAGA		

The threshold cycle values (C_t_) were first normalised to reference GAPDH mRNA (ΔC_t_) and the normalised mRNA levels of genes of interest were calculated as 2^(-ΔC_t_)^. The normalised mRNA levels of a particular cytokine were then used for the *t*-test comparison between the infected and non-infected birds. Finally, to display the fold induction after infection, 2^(-ΔΔC_t_) ^values were calculated for each cytokine mRNA levels by subtracting the normalised average C_t _of the gene of interest in the infected and non-infected chickens.

### Statistics and reproducibility

ANOVA with Tuckey's *post hoc *test was used for the analysis of bacterial counts and heterophil infiltration in infected chickens. The cytokine responses of chickens infected with the particular mutants and those of the non-infected controls were compared by the *t*-test.

## Authors' contributions

DK and AS constructed the SPI mutants, FS, HH, AMS and AI were responsible for the animal experiments. VK and BN analysed the samples by histology scoring and JV performed the cytokine expression by RT PCR. IR together with BN designed the experiments and wrote the manuscript. All authors read and approved the final manuscript.
